# Guiding officers to deflect citizens to treatment: an examination of police department policies in Illinois

**DOI:** 10.1186/s40352-023-00207-y

**Published:** 2023-02-08

**Authors:** Jessica Reichert, Sharyn Adams, Jirka Taylor, Brandon del Pozo

**Affiliations:** 1 Center for Justice Research and Evaluation, Illinois Criminal Justice Information Authority, 60 E. Van Buren St., Suite 650, Chicago, IL 60605 USA; 2 RAND Corporation, 1200 S Hayes St, Arlington, VA 22202 USA; 3 The Warren Alpert Medical School of Brown University, 593 Eddy Street, Providence, RI 02903 USA; 4 Rhode Island Hospital, 593 Eddy Street, 02903 Providence, USA

**Keywords:** Police, Policy, Deflection, Diversion, Substance use disorder, Treatment

## Abstract

**Background:**

The U.S. overdose crisis has motivated police departments to enact policies allowing officers to directly deflect individuals to substance use disorder treatment and other services shown to reduce recidivism and subsequent overdose risk, as well as refer people who voluntarily present at police facilities with a desire for treatment. As a new way of operating, and one that relies on an officer’s use of discretion for successful implementation, the practice benefits from guidance through written directives, training, and supervisory support. However, there is little information on the establishment, content, and execution of police department deflection policies, which hampers the implementation and dissemination of this promising practice.

We analyzed 16 policies of Illinois police department deflection programs. Using content analysis methodology, we coded the policies for language and terminology, as well as program components and procedures. We aimed to examine how the policies were written, as well as the content intending to guide officers in their work.

**Results:**

We found the policies and programs had notable differences in length, detail, terminology, and reading level. Only one policy mentioned the use of any type of addiction treatment medication, many used stigmatizing language (e.g., “abuse” and “addict”), and few mentioned “harm reduction” or training in the practice of deflection. Many policies restricted participation in deflection (i.e., no minors, outstanding warrants, current withdrawal symptoms), and critically, a majority of policies allowed police officers to exclude people from participation based on their own judgment.

**Conclusions:**

We recommend police departments consider the readability of their policies and reduce barriers to deflection program participation to engage a larger pool of citizens in need of substance use disorder treatment. Since there is limited research on police policies generally, and the field of deflection is relatively new, this study offers insight into the content of different department policies and more specifically, how officers are directed to operate deflection programs.

## Introduction

### Deflection as an emerging practice

Due largely to a U.S. opioid crisis of historic proportions and the failed War on Drugs, there have been calls to reform the way police respond to community members who use drugs (Cooper, 2015; del Pozo et al., 2021; Earp et al., 2021). One reform effort steadily growing in popularity is deflection, in which police officers make a warm handoff of people to behavioral health treatment or other services as an alternative to arrest (Charlier & Reichert, 2020; Enos, 2021; Lindquist-Grantz et al., 2021). While the operationalization of deflection programs varies across jurisdictions, they share the overarching objective of identifying individuals who could benefit from connections with treatment and other services and facilitating such connections. In doing so, deflection programs aim to improve outcomes pertaining to both public health (e.g., reduced substance use-related harms such as overdose) and public safety (e.g., the reduced justice involvement of people who use drugs) (Charlier & Reichert, 2020).

Owing to the relatively recent emergence of police deflection, its evidence base and best practices are nascent. In an attempt to capture the scope and breadth of deflection practices in the United States, a survey of 329 deflection programs in 39 states found the majority of programs started in 2018, featured police working in partnership with behavioral health treatment providers, and offered linkages to treatment and recovery support (NORC & Center for Health & Justice at TASC, 2021). A systematic scoping review of the evaluations of 31 deflection programs found limited, but promising, evidence for reduced recidivism, substance use, and improved psychosocial outcomes (Lindquist-Grantz et al., 2021). The first systematic review of deflection and post-arrest diversion, consisting of 37 studies, concluded that deflection was effective at preventing criminal recidivism, and held promise for improving health outcomes at reduced social and public safety costs (Blais et al., 2022).

One area of emerging deflection practice that has not received attention in the existing research are the specific policies guiding the practice of law enforcement officers. Many police departments have established deflection policies, but this growing body of official guidance has not been subject to formal examination. This paper aims to address this gap, with the aim of better understanding how law enforcement agencies formally organize and direct their deflection programs, which in turn can help inform efforts to identify best practices and promote their dissemination among other departments. This effort directly responds to an explicitly identified need for a “clear policy direction” for police for effective policing (International Association of Chiefs of Police & University of Cincinnati, 2020, p. 1) and for “more formalization of deflection practices through policy” (NORC & Center for Health & Justice at TASC, 2021, p. 21).

### The importance of policies for law enforcement agencies

A policy is an official feature of an agency’s institutional structure in the form of documented protocols, procedures, manuals, or laws (Sampson et al., 2013). As an indication of a police department’s commitment to professional conduct (Satula & Sparger, 2020; Stone & Travis, 2011), policies provide officers with concrete guidance and directives about the manner in which actions, tasks, and operations are to be performed (International Association of Chiefs of Police, n.d.), and provide officers with criteria and information to assist in making judgments and discretionary decisions. In addition to guiding action, sound policies can protect officers, managers, and municipalities from liability because they proactively informing officers about their duties and how best to perform them effectively, and in accordance with the law. For officers, policies can establish performance standards and consistency, as well as promote accountability, responsibility, and discipline (Carpenter, 2000).

In the past two decades, there has been significant innovation and reform in police practice (Brooks et al., 2020, Weisburd & Braga, 2006), with policy development serving as a critical lever in this effort. Critics of excessive force and discriminatory policing practices have called for reform and increased police oversight through the adoption of formal policies (Mummolo, 2017; Ponomarenko, 2019). The resulting policies have covered a wide range of domains: officer use of force (Gillispie, 2016; International Association of Chiefs of Police, 2020); deflection to behavioral health treatment (NORC & Center for Health & Justice at TASC, 2021); technology, such as body-worn cameras (McKesson et al., 2016: White & Fradella, 2018); and training, such as for improved mental health crisis response (Compton et al., 2010). As these innovations and reforms continue to evolve in the context of intense and ongoing scrutiny of police practice, police departments use their written policies to guide officers in the successful execution of new practices and show good faith efforts in improving policing to the public (Mummolo, 2017). In this vein, deflection, as another example of novel policing practice, stands to benefit from clear policy development and formulation.

This study adds to what is known about implementation science, as well as translational criminology, evidence-based policing, and policy implementation research (Laub, 2011; Nilsen et al., 2013; Sherman, 2013). Implementation science is the study of how research can guide effective implementation of programs and practices in an existing organization (Eccles & Mittman, 2006; Gleicher, 2017; Zielinski et al., 2020). Deflection often represents a new way of doing business for police departments, and in order to be successful and sustainable, the departments should prepare and support police officers and supervisors (Lerch et al., 2011; Weiner, 2009). Written policies outlining their deflection program and offering directives to officers to help facilitate successful implementation.

As these novel practices are more widely implemented, policies also play a critical role in guiding officer behavior. Police officers have much discretion in their decision making when out in the field (Alpert et al., 2004). Police-citizen encounters are complex and varied, which often requires on-the-spot decision making (Grattet, 2004). Officers may decide where to patrol and whom to stop, as well as choose to do nothing, talk to citizens, issue a warning, write a ticket, or make an arrest (Ponomarenko, 2019). Such discretion has the potential to produce disparities in how police interact with citizens (Nowacki & Spencer, 2019). As stated by the Michigan Commission on Law Enforcement Standards (2017), “decisions are made outside the view of supervisors and guidance through written protocols and best practices becomes even more crucial” (p. 2). Police policies can be one step to constrain or guide that discretion (Nowaki & Spencer, 2019).

Further, there is some, but limited, evidence that police policies can directly impact officer behavior. One study examined 2013 changes to New York Police Department Policies on “stop and frisk” which mandated officers document thorough narrative descriptions to justify stops of suspects (Mummolo, 2017). The author found those policies directly caused an increase in stops that produced evidence of criminal activity and decreased stops that produced no evidence. A study of police use of force policies also found restrictive policies led to officers using force less than officers with permissive policies (Terrill & Paoline, 2013). In a study of U.S. police deflection programs, police officers who were interviewed expressed that they may not have individually decided to support deflection, but were nonetheless following new deflection policies instituted by their police department (Taylor, 2023). Therefore, police policies, and more specifically deflection policies, can impact policing behaviors, suggesting the practice would benefit from a close examination of their content.

### Effective policy development

Effective police policies set expectations, provide guidance, and protect officers and agencies (Walker & Archbold, 2018). Existing literature has offered insights into ways to make policies effective. Stinchcombe (2001) theorized that for formal policies or rules to be effective, they must have the following components:

1. *Cognitive adequacy* – translate well into real situations to guide action.
2. *Communicability* – are transparent and easily understood by the intended audience.
3. *Trajectory of improvement* – allow for feedback and updating.

Therefore, policies should follow these components for successful implementation in police departments (Grattet, 2004). Department policies should be organized, coherent, and accessible to police officers (Carpenter, 2000). Further, policies should be written in terms of what offers *should* do, rather than what they *should not* do and take into account organizational culture and available community resources (Michigan Commission on Law Enforcement Standards, 2017). The person writing the policy, often an additional responsibility to their full-time assignment, must have strong written communication skills, and show attention to detail. Policies should be internally reviewed by officers in different positions (Orrick, 2018), as well as by external stakeholders and the public (Ponomarenko, 2019). Policies need to be kept up-to-date, reflect the best current practices, and be congruent with other policies across the agency (Lexipol, n.d.).

Some police departments share their policies and general orders online, demonstrating transparency and providing guidance for other departments. In 2015, 12 police departments in the 25 largest U.S. cities shared their policies with the public online (Ciaramella, 2015). There are organizations that provide police policy guidance, including the Legislative Analysis and Public Policy Association; the International Association of Chiefs of Police (which has a National Law Enforcement Model Policy Center); and state police chiefs’ associations (Orrick, 2018). Further, the U.S. Department of Justice encourages police policy reform through the provision of model police policies on a number of areas including training, investigations, data collection, and diversion (Motley Jr. et al., 2020). However, model policies should be more of a general guide than prescriptive of exact language to use as state and local laws, communities, and agencies vary by jurisdiction (Orrick, 2018).

An important corollary benefit to the trend toward publicly disclosing policies is their availability for review and analysis. Examining policies can shed light on what departments are doing (or at least claim to be doing, or aspire to do, and how policies are associated with various outcomes of interest). Against this backdrop, this study aims to examine policies pertaining to deflection programs operating in Illinois police departments. It adds to the literature on how police directives are written, and how their content can provide a way to guide and reform policing practices. Specifically, this paper sets out to address the following research questions:

- To what extent are police policies on deflection understandable to police officers in terms of reading ease and grade level?
- What terminology is used in police policies to describe addiction, substances, medications to treat substance use disorders (SUDs), and deflection program components?
- What differences and consistencies emerge from a comparison of the policies under study?
- What are the deflection program procedures outlined in police policies including stakeholders, services offered, and eligibility criteria?

## Methods

We conducted a content analysis of Illinois police department deflection policies. We contacted the Chiefs of police whose departments had deflection programs to obtain their departmental policies on deflection, 22 in all. These included 12 municipal police departments in Lake County, Illinois that operate the A Way Out deflection program, one of six sites in the country selected as a part of a larger [name deleted to maintain the integrity of the review process] study funded by National Institute of Justice. In addition, we contacted an additional six departments funded through federal or state grants through the [name deleted to maintain the integrity of the review process], and four additional programs of which we were aware. We sent an initial email to the Lake County departments in April 2022 and a reminder email in May 2022. We sent an email to the additional programs in June and July 2022. Of the 22 departments, 16 sent their policies, for a 72.7% response rate.

We performed a content analysis of the police department policies regarding deflection programs. A content analysis is a research tool to quantify the use of certain words, themes, and concepts in qualitative data (Neuendorf, 2017). Content analysis allows for the examination and comparison of policies across departments (Eyler, et al., 2010; Hall & Steiner, 2020; Legislative Analysis and Public Policy Association, 2021a; McKesson et al., 2016; Zardo & Collie, 2014).

To analyze the readability of the policies under study, we used a Microsoft Word feature to measure word count and readability statistics (Microsoft, n.d.). Readability was measured using the Flesch Reading Ease and Flesch-Kincaid Grade Level based on sentence length (average number of words in a sentence) and word length (average number of syllables per word). The Flesch Reading Ease score provides the score of a text from 0 to 100 with 0 to 40 as “very difficult” to “difficult” reading and 80 and above as “easy” to “very easy” (Flesch, 1981; Stockmeyer, 2009). The Flesch-Kincaid Grade Level provides a U.S. grade level of education required to understand a text with an 7th or 8th grade reading level recommended for most public documents (Marchand, 2017). Then, for interrater reliability, two researchers independently coded the policies on coding sheets. Discrepancies were discussed and resolved to create one consolidated coding sheet per policy (Table 1). We coded policy contents as dichotomous yes/no if the policy contained certain terms or elements (see Tables 3, 4, and 5), so counts are mutually exclusive and not the number of times terms were used or referenced. We then transferred the policies to Nvivo 12 qualitative software to analyze the text by themes. The study was not submitted for IRB review because it was not human subjects research.

**Table 1:** Police Department Deflection Polices Coding Sheet Categorical Descriptions

Category	Description
General program information	Police agency/department, program name, program type/pathway
General policy information	Effective date, revision date(s), total number of words, reading level
Terminology used	Terms used related to substances and substance use disorder, program type
Stakeholders and partners	Are any mentioned as participating in the program: EMS, fire department, sheriff’s department, prosecutor, health department, treatment providers, recovery coaches, volunteers?
Eligibility	Are eligibility criteria established? Who is excluded?
Voluntary	Is there mention that the program is voluntary for citizens?
Intake procedures	Are intake procedures established?
Assessment of clients	Is there assessment for substance use disorders? Who completes them?
Transportation of clients	Are clients transported by officers or others?
Confiscation of drugs or paraphernalia	Will officers confiscate these items with no arrest?
Training of officers	Is the training of officers mentioned?
Research and data management	Is there mention of anything about data collection, analysis, reporting?

### Sample of policies

Table 2 shares information about the participating police departments, programs, and the date each policy was established. The earliest policy was established in 2016, and the latest in 2022. Fourteen programs employed the self-referral deflection pathway, in which citizens voluntarily initiate contact at a police department for a referral to treatment and /or services. Two, the Kane County Sheriff’s Department and the Chicago Police Department, used an officer intervention model where engagement is initiated by the officer, either alone or as part of a co-response team (Charlier & Reichert, 2020; Schiff et al., 2017).

**Table 2:** Illinois Police Departments Policies Included in Study

Program Name	Police department name	City or county program	Urban/ rural	Date policy established
Community Addiction and Recovery Effort (CARE)	Arlington Heights PD	City	Urban	7/26/2018
Deflection Program	Schiller Park PD	City	Urban	2/20/2022
Elk Grove Village Cares	Elk Grove Village PD	County	Urban	12/29/2021
Kane County A Way Out	Kane County Sheriff	County	Urban	–
Lake County A Way Out	Antioch PD	County	Urban	9/9/2019
	Deerfield PD	County	Urban	9/28/2018
	Fox Lake PD	County	Urban	8/9/2017
	Grayslake PD	County	Urban	6/2/2016
	Lake Zurich PD	County	Urban	5/1/2018
	Libertyville PD	County	Urban	5/24/2016
	Mundelein PD	County	Urban	5/23/2016
	Round Lake Beach PD	County	Urban	6/4/2021
	Wauconda PD	County	Urban	9/12/2019
Mokena Safe Passage	Mokena PD	City	Urban	–
Narcotics Arrest Diversion Program	Chicago PD	City	Urban	5/17/2022
Taylorville Safe Passage	Taylorville PD	City	Rural	6/14/2022

Sample of 16 police department policies. *PD* Police department. Two policies did not indicate date policy was established

## Results

### Readability and terminology

Ten departments referred to their deflection policies as “general orders” or “orders;” three used the term “directives,” and four used the term “policies,” with Kane County using both “policies” and “directives.” In policing, general orders are broad groupings of basic rules and regulations, while directives and policies are subject-specific, detailed, and highly interchangeable as terms. The mean word count of police department deflection policies was 1034.5, ranging from 517 to 1790 words. We found the Flesch Reading Ease score of the police policies had a mean of 38.9, ranging from 21.4 to 43.7. Scores less than 40 indicate difficult to very difficult reading. We found the Flesch-Kinkaid Reading Level was on average readable for grade 13.2, with a range of grade level from 11.7 to 16.

Table 3 shares counts of terms and phrases used in the police department deflection policies. A majority of police department polices used the term “addiction” and “overdose” when discussing SUDs. A majority of departments used the terms “substances,” “prescriptions,” and “narcotics/illicit drugs.” Almost all the policies used the term “treatment,” but a small proportion of policies noted the terms of “recovery.” One agency used the term “harm reduction,” defining it as “a range of public health policies designed to lessen the negative social and/or physical consequences associated with unmanaged substance use, mental illness, and/or being unhoused.” The policy further indicated one of the core principles of the program was to “improve public safety and public health through research-based, health-oriented, and harm reduction interventions, when appropriate.”

**Table 3:** Terminology Used in Police Deflection Policies

Word/ phrase used	Number of department policies	
	*n*	%
Description of heath condition		
Addiction	10	62.5
Overdose	10	62.5
Abuse	7	43.8
Substance use disorder	8	50.0
Addict	4	25.0
Dependence	0	0.0
Opioid use disorder	0	0.0
Medications for opioid use disorder		
Naloxone/Narcan®	2	12.5
Methadone	1	6.3
Buprenorphine/Suboxone®	0	0.0
Naltrexone/Vivitrol®	0	0.0
Substance terms		
Substance	13	81.3
Prescription	1	6.3
Drug	13	81.3
Narcotics or illicit drug	9	56.3
Opioid/opiate/heroin	8	50.0
Alcohol	5	31.3
Stimulant, methamphetamine/meth	2	12.5
Cannabis	0	0.0
Program description/components		
Treatment	15	93.8
Recovery	4	25.0
Diversion	3	18.8
Deflection	2	12.5
Harm reduction	1	6.3
Warm handoff	0	0.0

Based on review of 16 police department deflection policies

### Program procedures

Four department policies directed the use of intake forms, and 13 required written consent for program participation. Most departments considered people with outstanding arrest warrants ineligible for the program, but two policies indicated they would attempt to resolve warrants through coordination with local prosecutors. Thirteen policies provided formal eligibility requirements for program participants. Table 4 indicates the frequency of reasons for ineligibility. Except for being underage or lacking parental consent, the most frequent exclusion criteria, used by nine departments each, were a need for medical assistance, exhibiting symptoms of withdrawal, and the belief of an officer that deflection was inappropriate in the case at hand.

**Table 4:** Reasons for Program Ineligibility

Reason for ineligibility	Number of department policies	
	*n*	%
Subject underage, lacks parent/guardian consent	10	62.5
Subject presenting symptoms of withdrawal or needs other medical treatment	9	56.3
Officers have reasonable belief that subject should be ineligible	9	56.3
Has an outstanding warrant	8	50.0
3+ prior drug charges *or* 1 intent to distribute charge	7	43.8
3+ prior drug charges *and* 1 intent to distribute charge	2	12.5
Current additional charges	3	18.8
Lacks legal identification	1	6.3
Prior non-drug offense	1	6.3
Reason to believe the contraband was going to be sold	1	6.3

Based on review of 16 police department deflection policies

Two departments mentioned training officers to participate in deflection programs, without discussing the curriculum or providing other details. One policy stated that the department’s Special Victims Unit’s sergeant would coordinate training. Table 5 provides detail on deflection program stakeholders and how participants are transported to SUD treatment based on policy text. A majority of policies mentioned treatment providers, hospitals/ERs, or health departments as stakeholders and majority of policies had officers providing the transportation of participants to SUD treatment.

**Table 5:** Deflection Program Stakeholders Named in Department Policies

Stakeholder	Number of department policies	
	*n*	%
Program stakeholders		
Treatment provider	13	81.3
Hospital/emergency room	12	75.0
Health department	10	62.5
Prosecutor	7	43.8
EMS	4	25.0
Fire department	4	25.0
Jail	2	12.5
Sheriff/ sheriff’s department	1	6.3
Transportation provider		
Officer	13	81.3
Ambulance if needed	5	31.3
Friend or family	3	18.8
Volunteer	3	18.8
Other	1	6.3

Based on review of 16 police department deflection policies. Transportation providers drive program participants to substance use disorder treatment

## Discussion

### Policy readability and language

Given that deflection programs are an emerging practice that have yet to gain widespread adoption and support among agencies and officers, it is critical that the policies directing the practice be clear, concise, and easy to understand and follow. In contrast, based on their readability scores, the deflection policies studied would be considered difficult to read for a general audience (Stockmeyer, 2009). We found the policies were, on average, readable by a college freshman, but many departments require only a high school diploma or equivalent (U.S. Bureau of Labor Statistics, n.d.) and one study found 1.3% of U.S. police departments require a 4-year degree (Gardiner, 2017). Considering that many more officers have some college rather than a full four-year degree, these policies may be understandable to most, but potentially not all, officers. To be useful and effective, policies should be easily understood by the officers expected to adhere to them (Carpenter, 2000; Grattet, 2004; Stinchcombe, 2001). Police departments should run readability tests and allow for officer review and comment before being finalized. Orrick (2018) recommended policies be reviewed by many individuals including sworn and civilian police staff, legal staff, or through a designated policy committee. In addition, policies can be reviewed by external stakeholders and subject matter experts, as well as be made available for public comment (Ponomarenko, 2019). However, readability may be affected by the use of proper clinical and scientific terminology used to define health conditions (e.g., substance use disorder) (Kelly et al., 2016).

We found that many policies used stigmatizing language. Seven policies used the term “abuse” related to substance use, and four policies used “addict,” which are terms that have been shown to increase negative attitudes toward those who use drugs (Wakeman, 2019). The National Institute on Drug Abuse recommends not using “abuse” but “use” and not using “addict” but “person with substance use disorder” (National Institute on Drug Abuse, n.d.). The use of these terms in police policies for deflection programs meant to assist persons with SUD can undermine, and potentially reduce the effectiveness of, those programs (Kelly et al., 2016). Therefore, police departments should use appropriate terms that reduce stigma and negative bias in all policies and communications.

In addition, “harm reduction” was only referenced in one policy, which may indicate that it is not a tenet of the deflection programs studied. This is supported by findings of the national deflection program survey, in which 43% of police respondents indicated needing harm reduction training (NORC & Center for Health & Justice at TASC, 2021). At present, such curricula have yet to be extensively developed, implemented, or evaluated (Khorasheh et al., 2019), although emerging evidence from domestic and international settings suggests framing police-led harm reduction interventions in terms of officer wellness and occupational safety can enhance their acceptability and speed their implementation (Baker et al., 2022; Davis & Beletsky, 2009).

Along similar lines, “naloxone” was explicitly mentioned in only one document, although it is possible programs participate in naloxone distribution without addressing it in their deflection policies. In addition, most policies listed health departments as a stakeholder, so policies may assume harm reduction practices are supported elsewhere or police harm reduction practices are noted in separate policies (Yatsco et al., 2020). Harm reduction can be an additional tool beyond, or in addition to, treatment with interventions to reduce the negative effects of substance use (e.g., naloxone to reverse overdose, syringes to prevent infections due to needle sharing) (Logan & Marlatt, 2010). Further, it is possible that some presenting clients may ultimately decide not to engage with treatment services or suitable services may not be available. In such situations, connection with harm reduction resources can play an important role mitigating the risk of acute harms and staying engaged with the client until they are connected with a treatment service. Therefore, police may want to include referrals to service providers or local health departments that offer harm reduction services. Community overdose education and nasal naloxone distribution programs have been shown to reduce overdose (Walley et al., 2013) and police working in deflection programs can and should incorporate the use of naloxone to save lives (Fisher et al., 2016; Lowder et al., 2020). Further, police deflection programs using a harm reduction approach, helping those who may be actively using drugs, have been found to be effective at reducing arrests, increasing employment, and improving housing (Clifasefi et al., 2017; Collins et al., 2015; Diriba & Whitlock, 2022; Firesheets et al., 2022; International Association of Chiefs of Police & University of Cincinnati, 2020; Morrissey et al., 2019; Perrone, et al., 2022)..

Just two departments explicitly touched on training in their deflection program policies, which may indicate that officer training is not a mandated component of deflection. This is supported by the national deflection program study, which found that about one-third of programs had specialized deflection training and of those, half had allotted four or fewer training hours to the subject (NORC & Center for Health & Justice at TASC, 2021). Police academies initially teach department policies and procedures, but police officers need continuing education on emerging best practices and changes in department policies (Walker & Archbold, 2018). Implementation science identifies training as a major component to successful initiation of a program or intervention (Kirchner et al., 2014; Taxman & Rudes, 2013). Training should comprehensive and interactive and provided at the start of the program and should be continued throughout the life of the program (Bertram et al., 2015; Fixsen et al., 2009; Gleicher, 2017). Police officers working to deflect individuals with SUD should have some baseline understanding of related topic areas to effectively do the work (Barberi & Taxman, 2019; Branson, 2016; Charlier & Reichert, 2020; Ekelund & Charlier, 2019; Reichert et al., 2017; Reichert et al., 2023). Law enforcement needs clear policies and training to be effective in both crisis situations and more routine activities and interactions with people with a behavioral health condition (International Association of Chiefs of Police & University of Cincinnati, 2020). Additionally, incentives should be provided to officers to complete training (Blandford, 2021), as well as refresher courses to support knowledge and address implementation barriers (Skorek & Devitt Westly, 2015).

### Program eligibility

The policies we examined stipulated many reasons why a person would be ineligible for deflection. However, if deflection programs are meant to assist community members with SUD get treatment, police departments should explore barriers that could be removed and not make absolute exceptions in writing in policy. We found 10 policies did allow underage youth to be eligible for deflection and referral to SUD treatment. In the United States, the National Survey of Drug Use and Health (NSDUH) estimates that more than 1 million youth aged 12–17 need illicit drug treatment, yet less than 3% receive it (U.S. Department of Health and Human Services, Substance Abuse and Mental Health Services Administration, Center for Behavioral Health Statistics and Quality, 2021). Further, this may underestimate the true extent of treatment need in the country given the survey’s limitations regarding use prevalence estimates (Midgette et al., 2021; Reuter et al., 2021). States vary in their laws on consent for minors to participate in SUD treatment, which may be required from a parent, the minor, or not specified (Kerwin et al., 2015; Lallemont et al., 2009). In Illinois, whose policies were examined in this study, state law allows for minors aged 12 or older to have decision-making authority for inpatient and outpatient SUD treatment (Kerwin et al., 2015). Therefore, departments should examine if state law prohibits minors consenting to treatment. Research has found youth with SUD and other mental disorders can benefit greatly from behavioral and family therapy (Brewer et al., 2017).

It is critical to note that nine policies specifically empowered police officers to decide on their own if deflection is appropriate, by using discretionary judgment based on a reasonable belief that subject should be ineligible This accords with findings that police often feel they have discretionary control over decisions to arrest people for nonviolent misdemeanors and drug offense (del Pozo et al., 2021), but broaches important questions in doing so. Given police discretion in deciding the course of encounters with people who use drugs, research should investigate what factors shape the use of this discretion, and what policies, training, and cultural factors can best direct it toward evidence-based decisions that improve health and justice outcomes. If police officers do not fully comprehend the effectiveness of medications for opioid use disorder in reducing both recidivism and overdose, for example, then they are missing a key piece of evidence in making their assessments. In that vein, policies that encourage the use of judgment and discretion should provide substantive guidance about what factors to consider, and the relative benefits and concerns about both arrest and deflection.

The discretion that police have specifically been given in these policies also suggests researchers need to understand and mitigate the role stigma plays in the way police interact with people who use drugs. Prior research has found police discretion may be shaped by many factors including officer age, race, and rank (Beyer et al., 2002), police culture (Barberi & Taxman, 2019), suspect characteristics and demeanor, and the nature of the offense (Brown et al., 2009; Engel, et al., 2018; Novak & Engel, 2005). Studies have found that police believe people who use drugs will not hesitate to lie, lack willpower, are dangerous to be around, and cannot be trusted in situations such as child care (del Pozo et al., 2021; Kruis et al., 2020; Kruis & Merlo, 2020); in general they evince the stigma toward people who use drugs found in the general population.

While some of these beliefs are broad, inaccurate stereotypes, and others are a product of the consequences of addiction when true, such stigma is harmful in two ways: it may lead police to believe it is not worthwhile investing time and resources in a person who uses drugs beyond arresting them, and when internalized by people who use drugs themselves, it may prevent them from seeking help in police setting out of shame and fear. This latter barrier is especially likely for departments engaging in the self-referral deflection pathway, the predominant model studied here, which relies on people who use drugs to take the step of voluntarily entering into contact with police. There is also evidence that if unchecked, stigma leads first responders to discount the value of the most effective treatments for addiction (Kruis et al., 2021). To guard against the hazards of stigma, policies that offer guidance to minimize it hold particular promise. For example, policies that direct officers not to handcuff people undergoing the deflection process, or to house and process them among the population of general prisoners can send important signals that substance use is not a crime per se, and the intended response isn’t a punitive one. Some of the policies in this study include such guidance, although it was not systematically present.

Eight policies stated individuals with outstanding warrants were ineligible for deflection programs. Nationally, deflection programs vary on how they deal with arrest warrants. For example, Kentucky and New Jersey deflection programs exclude those with outstanding warrants (Legislative Analysis and Public Policy Association, 2021); however, at least one Illinois program allows officers to collaborate with prosecutors to resolve warrants if possible (Kopak & Gleicher, 2020). Police departments should collaborate with local prosecutors on the ability to resolve warrants, while taking into consideration the nature and severity of the offense that led to the warrant. Criminal justice stakeholders such as judges, prosecutors, and community correction officers, can help remove impediments to deflection participation (NORC & Center for Health & Justice at TASC, 2021).

Our analysis indicated that a majority of police departments in our sample did not admit individuals with prior charges, especially those for drug offenses. This is problematic for several reasons. First, such rules may render a notable share of potential clients ineligible; to illustrate, an analysis by Kilmer et al. (in press) showed that the annual nationwide number of arrests for opioid-related offenses “might be in the range of 200,000-300,000 annually” and the annual number of arrests to which opioid use could be linked (e.g., property crimes) could plausibly be “on the order of 1 million.” Second, it perpetuates the stigma associated with a past drug offense beyond the duration of the sentence and may thus reinforce the reluctance to seek help among people who use drugs. Third, introducing conditions on eligibility, particularly if not clearly delineated (e.g., resolving outstanding warrants on an ad hoc basis), may create barriers to entry, especially for programs that require clients to trust the police that there would be no negative repercussions from coming forward. Finally, excluding individuals with prior charges may be a missed opportunity to divert individuals who may be at higher risk for offending due to substance misuse (Timko et al., 2017). According to the Risk, Need, and Responsivity (RNR) model, which has strong empirical support, those with high risk and needs should have responses providing targeted and effective interventions, such as substance use disorder treatment (Bonta & Andrews, 2007; Prendergast et al., 2013; Wormith & Zidenberg, 2018). For these reasons, police departments may want to revisit their eligibility rules and reconsider which ones are truly necessary for the program’s successful operation.

We found nine policies excluded potential participants exhibiting symptoms of withdrawal, which occurs when ceasing or reducing use of a drug when physically dependent. Although the reasons for excluding individuals who are in withdrawal are unknown, reducing impediments and establishing protocols for screening and referral for management of withdrawal and follow-up are needed (Bernstein & D’Onofrio, 2017; Kosten & O’Connor, 2003). Given the potential of medications for opioid use disorder (MOUD) to address withdrawals, it is therefore noteworthy that policies studied did not touch on linkage to treatment with MOUD in an explicit way, discuss the role of these medications in deflection, or as one of the goals of the policy. Although nearly all policies generally mentioned “treatment,” just one policy mentioned a medication—methadone. A growing body of evidence spanning three decades has shown that treatment with medications for opioid use disorder reduces crime and re-arrest (Ball & Ross, 2012; Evans et al., 2022; Evans et al., 2019), while saving lives (National Academies of Sciences Engineering & Medicine, 2019; Santo Jr et al., 2021). At the same time, arrest and subsequent incarceration, which induces and prolongs withdrawal, has been consistently shown to dramatically increase risk of fatal overdose upon release (Binswanger et al., 2011; Binswanger et al., 2013; Finlay et al., 2018; Ranapurwala et al., 2018). Police departments that envision deflection as an opportunity to both reduce the crimes resulting from addiction and save lives from overdose should emphasize to rank and file officers that among the practice’s critical benefits are the opportunity for a linkage to medications for opioid use disorder (as well as other services and resources), while foregoing the acute overdose risks of arrest and incarceration. That nine policies excluded potential participants exhibiting symptoms of withdrawal highlights that they have yet to incorporate the best available evidence. Arrest during a period of withdrawal is extremely dangerous for prisoners, while linkage to medication could be a step toward ameliorating not only such withdrawal (Pergolizzi Jr et al., 2020), but also the underlying addiction, which often motivates criminal behavior.

For people using opioids, police deflection programs which are currently not doing so should consider referral to a physician who can prescribe medications (Anglin et al., 2013), or to alternate means of withdrawal management for people using other substances. Doing so can assist in displaying empathy and building trust with community members, who may go on to become deflection program clients (World Health Organization, 2009). Departments should also fully educate officers on the role and importance of MOUD and write policies that highlight it as a critical tool given the stated goals of a deflection program.

### Study limitations

Our study examined 16 police deflection policies but was limited to Illinois programs that were largely homogenous. Fourteen were self-referral programs, in which a citizen initiates contact with police for help, and nine were a part of one county’s program (A Way Out). Therefore, there are limitations to generalizability. In addition, we examined written policies, but there may be informal procedures, or ones that exist as a separate program manual, rather than official department policy, of which we would be unaware. We also did not compare deflection policies to other policies in terms of readability and length, so we cannot say if concerns about readability pertain to deflection policies in particular, or are a more general concern.

It is also important to note we did not study the extent to which these policies were implemented with fidelity, or the extent to which officers utilized them. It is possible for a deflection program to exist that is rarely, if ever, utilized, but we have not studied the feasibility and acceptability of the policies we examined. Future research should do so, since lack of uptake would reflect a serious deficit in a policy enacted in response to an acute public health and safety crisis.

## Conclusions

We examined 16 police departments that had established official written policies governing their use of deflection. We examined these policies in terms of readability, language used, and specific rules and procedures. While police efforts to link people with SUD to treatment are generally preferable to arrest, this study suggests areas for improvement of the policies that direct these efforts. A main concern was the use of stigmatizing terminology, which should be removed from policies and the police lexicon and discouraged from use in the police vernacular. Second, the policies had little mention of officer training or harm reduction, which should be important components of successful police deflection programs. We found many police policies provided a long list of exclusions of persons from obtaining deflection. Many of the exclusion criteria (e.g., based on age, outstanding warrants, or exhibiting withdrawal symptoms) should be reassessed and potentially removed from policies in order to better contribute to population-level efforts to reduce addiction and overdose. This study examined policy content, but future research could explore officer perspectives on department policies, and the extent to which officers are aware of, and follow, those policies.

## Data Availability

The data used and analyzed during the current study were derived from police department policies and are available from the corresponding author on reasonable request.
